# The Use of Electronic Personal Health Records to Improve Medication Adherence and Patient Engagement: A Randomized Study of Non-valvular Atrial Fibrillation Patients

**DOI:** 10.19102/icrm.2017.080803

**Published:** 2017-08-15

**Authors:** Yu-Chieh Chen, Amelia E. Roebuck, Areej Sami, Özlem H. ERSIN, Michael J. Mirro

**Affiliations:** ^1^Department of Pharmacy, China Medical University Hospital, Taichung, Taiwan; ^2^Parkview Research Center, Parkview Health System, Fort Wayne, IN; ^3^Indiana University School of Medicine, Indianapolis, IN; ^4^College of Health and Behavioral Studies, James Madison University, Harrisonburg, VA

**Keywords:** Anticoagulant, atrial fibrillation, medication adherence, patient engagement, personal health records

## Abstract

Embolic stroke is a major complication of atrial fibrillation (AF) that frequently results in disability or death. The administration of oral anticoagulation can reduce stroke risk in AF patients; however, medication non-adherence can eliminate this benefit. To date, reported patient adherence rates to oral anticoagulation regimens vary. The objective of the current study was to examine the impact of medication-specific education delivered via a personal health record (PHR) system on medication adherence. A randomized, prospective study was conducted from February 2014 to June 2014 at Parkview Health, a not-for-profit, community-based health care clinic that serves a northeastern Indiana population of more than 820,000. AF patients receiving dabigatran (Pradaxa^®^; Boehringer Ingelheim GmbH, Ingelheim am Rhein, Germany) to prevent stroke participated in this study. The study participants were predominantly Caucasian males over 65 years of age who were educated, insured, and living above the poverty level. Patients were allowed to view online, download, and transmit health information via a PHR. The intervention group received PHR training and dabigatran education via the PHR. The control group received standard care and PHR access without training. A longitudinal survey pertaining to medication knowledge, medication adherence, and patient engagement was administered at baseline and at the end of the study. Medication-dispensing data collected from pharmacy refill prescriptions were used for calculating the medication possession ratio (MPR). Ninety patients were included in this study, and were randomly assigned to either the intervention group (n = 46) or the control group (n = 44). All participants completed the baseline survey, and 95.6% of patients finished the follow-up survey. The mean score for knowledge increased significantly in the intervention group (from 3.77 to 4.23, p = 0.005), but not in the control group (from 3.70 to 3.95, p = 0.72).

The MPR was significantly higher in the intervention group (97.47% vs. 87.67%, p = 0.001). Both groups had similar levels of improvement in Patient Activation Measure scores (from 63.0 to 65.8, p = 0.078 vs. from 63.1 to 63.6, p = 0.814). Patients who used the PHR achieved greater medication knowledge, resulting in improved medication adherence. To our knowledge, no published randomized trial has reported on the use of PHRs to improve medication adherence and knowledge. This study is the first to demonstrate a positive impact on anticoagulation adherence with PHR use.

## Introduction

Atrial fibrillation (AF) is a heart rhythm disorder affecting over three million Americans that increases the risk of stroke, with one in five strokes attributed to AF.^1^ Embolic strokes associated with AF are often fatal, and patients who do survive tend to be disabled in some fashion and are more likely to suffer a recurrence than patients with stroke resulting from other causes.^2^

### Management of AF and stroke

The use of oral anticoagulants (OACs) has been shown to effectively decrease stroke incidence by over 50%.^3^ Warfarin (Coumadin^®^; Bristol-Myers Squibb, New York, NY, USA) has been the predominant OAC prescribed for stroke prevention in patients with non-valvular AF.^4,5^ Its disadvantages, however, include a narrow therapeutic index, a potential for interaction with other drugs, and the need for frequent monitoring of international normalized ratio. Dabigatran etexilate (Pradaxa^®^; Boehringer Ingelheim GmbH, Ingelheim am Rhein, Germany) was the first direct OAC approved by the United States (US) Food and Drug Administration in 2010 to reduce the risk of stroke in AF patients.^6^ This direct thrombin inhibitor presents less complexity in how it is prescribed as compared with vitamin K antagonists, and has emerged as an alternate therapy to warfarin. In comparison with warfarin use, studies have shown that treatment with dabigatran results in lower rates of ischemic stroke and intracranial hemorrhage, but that it is also associated with a significant increase in major gastrointestinal bleeding.^7^ Although patients on a dabigatran regimen do not require routine monitoring, there are concerns associated with its use, including: (1) the limited information and guidance available as to its interactions with other drugs^8^; (2) less frequent patient visits with clinicians, resulting in a reduced amount of education; (3) a potential for reduced anticoagulation with intermittent medication adherence; (4) the proliferation of frequent public announcements in the media regarding bleeding risks, resulting in patient fear; and (5) a potential for diminished adherence to a twice-daily dosing regimen, specifically.

### Medication adherence

In a 30-day study of adherence to dabigatran use after orthopedic surgery, 31% of patients did not initially remember to resume their medication regimen, and 15% became non-compliant by taking more doses to compensate for their missed doses.^9^ One study from the US Veteran’s Administration hospital system database analyzed dabigatran adherence, finding that 28% of patients were non-adherent, and that lower adherence was associated with an increased risk for combined all-cause mortality and stroke.^10^ Another study found that anticoagulant clinic patients were more likely to have medication possession ratio (MPR) values of ≥80% (considered to be good adherence) on dabigatran at the end of a three-month period than the usual care group.^11^ In spite of existing dabigatran research, adherence for this new anticoagulant agent varies among studies and remains sparsely documented.

### Health information technology

In 2009, the American Recovery and Reinvestment Act established subsidiaries to foster the rapid adoption of health information technology, with the requirement that the technology used achieved or met Meaningful Use (MU) standards.^12^ Implementation of electronic personal health records (PHRs) was facilitated by the MU program in order to increase patient access to health information and improve communication between patients and health care providers—a key occurrence to MU. A PHR is an Internet-based tool that allows for patients to access and coordinate their health information. The National Academy of Medicine has identified the use of information technology as one of four critical factors in the improvement of health care delivery in the US.^13^ Medication management support via PHRs may be an effective means of promoting medication adherence in a specific patient population over a sustained period of time.^14,15^ However, studies demonstrating the use of PHRs for medication management remain lacking. Common barriers to medication adherence include poor patient–health care provider communication, inadequate patient knowledge about medications, low health literacy, patient skepticism of the need for treatment, fear of adverse drug effects, and patients’ experiences of symptom-free periods.^16,17^

### Objectives

That being said, the increased understanding of health conditions and medication knowledge should influence patients to adhere to their medication regimens. Additionally, more frequent bi-directional communication between patients and health care providers, aided by electronic communication, could reinforce patients’ adherence to their treatment plans. Presently, however, no published randomized trial has reported on the use of PHRs to improve patient medication adherence and knowledge following the introduction of the requirement of meeting MU at US health care institutions. Thus, this study focused on improving the understanding of PHR use on medication management. The study consisted of AF patients receiving anticoagulant medications to prevent stroke, with the goal of examining the impact of PHR-facilitated medication education on adherence, knowledge, and patient engagement.

## Methods

### PHR selection

The PHR used for this study was MyChart^®^ (Epic Systems Corp., Verona, WI, USA), and was available to all patients receiving care at Parkview Health System in Fort Wayne, IN. MyChart^®^ (Epic Systems Corp., Verona, WI, USA) follows Health Insurance Portability and Accountability Act (HIPAA) guidelines, and is accessible to patients via any Internet-connected computer or mobile device. This PHR is tethered to the enterprise electronic health record system, allowing for health care providers to view patient messaging while giving patients controlled access to the same Epic Systems Corp. (Verona, WI, USA) medical records used by their physicians. The self-serve functions allow for patients to manage aspects of their health and facilitate communications with physicians and health care providers. Patients can review test results, view upcoming and past appointments, schedule appointments, request prescription refills, and access educational resources related to health and wellbeing.

### Study population, setting and recruitment

**Figure 1:**
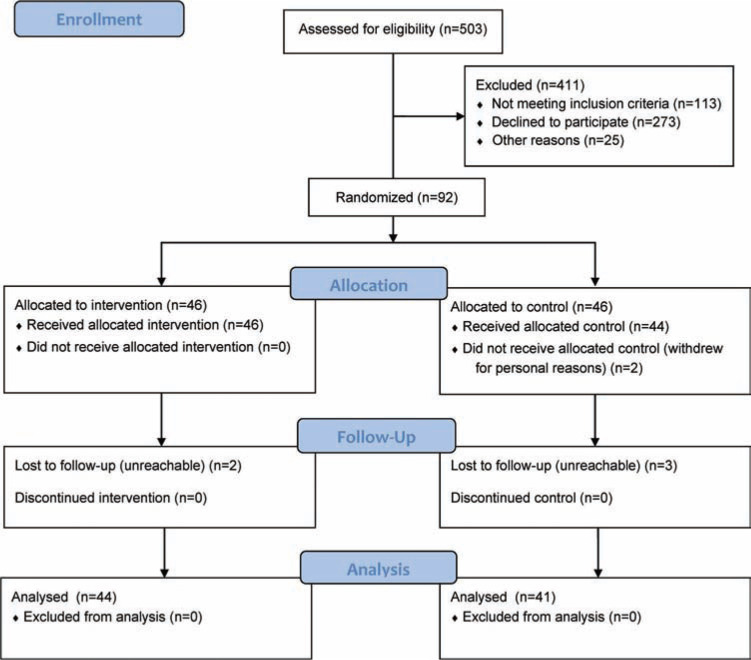
Consolidated Standards of Reporting Trials (CONSORT) flow diagram for study recruitment and follow-up.

This study’s setting was a cardiac clinic that conducts 80,000 office visits per year. The study population included adult patients (≥ 18 years of age) with AF identified through a chart review completed using electronic medical records. Inclusion criteria were (1) a diagnosis of AF, (2) receiving dabigatran for the prevention of stroke, (3) the ability to read and understand English, and (4) having access to the Internet. Exclusion criteria were (1) receiving warfarin, (2) being institutionalized for any reason, and (3) having a lack of physical or cognitive ability to carry out the tasks necessary for utilizing the PHR. Subject recruitment and follow-up are summarized in **Figure 1**. All of the patients (n = 90) included in this study were provided with a MyChart^®^ (Epic Systems Corp., Verona, WI, USA) account. This research was conducted as an unblinded, randomized, parallel controlled trial.

**Figure 2:**
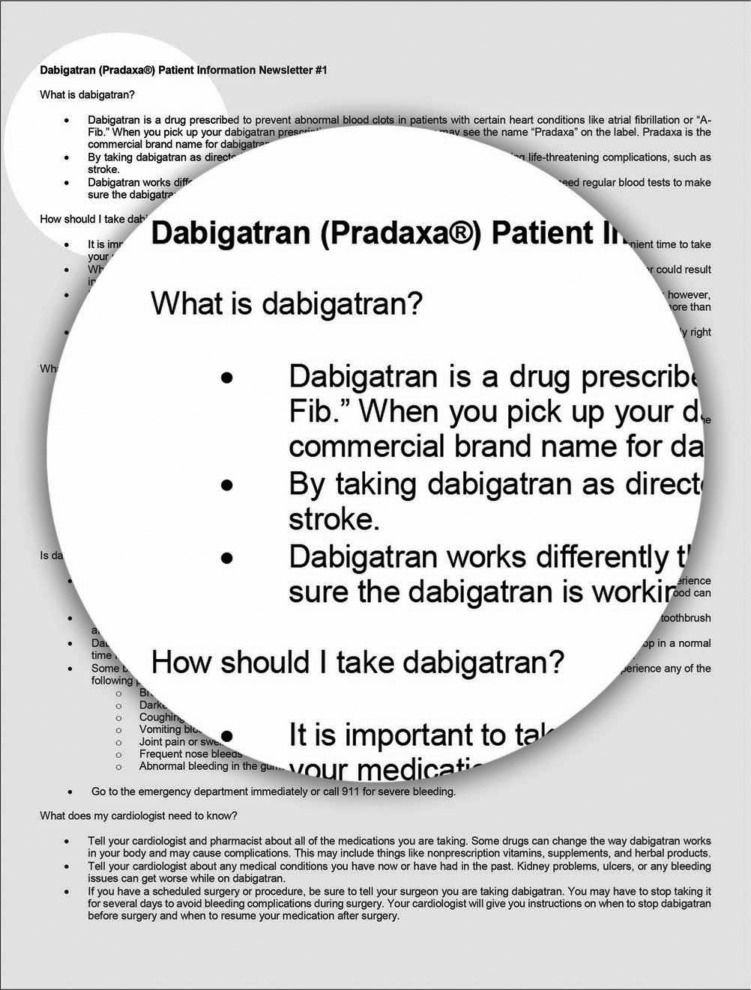
An example of a patient education newsletter distributed to the intervention group (n = 46) throughout the study period.

In this study, consenting patients were randomly assigned (1:1) via simple randomization to either the intervention group or to the standard care (control) group. The participants who were assigned to the intervention group experienced customized PHR interventions, including one-on-one training in PHR use, medication education material, and provider support. Previous studies have demonstrated that training patients is a cost-effective method for increasing their ability to use a PHR, which can lead to an increase in their confidence level in making health-related transactions, with finding health-related information, and with interacting with health care providers via the PHR.^18,19^ The training for the intervention group included logging into and navigating the MyChart^®^ (Epic Systems Corp., Verona, WI, USA) interface; using the secure messaging system; and viewing the available medication educational material, which consisted of newsletters prepared by a drug information specialist sent to patients’ MyChart^®^ (Epic Systems Corp., Verona, WI, USA) accounts at four, six, and 10 weeks, respectively, post enrollment. An example of the medication educational material disseminated can be found in **Figure 2**. At approximately eight weeks from the date of enrollment, patients in the intervention group were offered optional refresher training on MyChart^®^ (Epic Systems Corp., Verona, WI, USA) use. Notably, health care provider support was essential to the success of the intervention. Two research coordinators communicated with patients regularly via the MyChart^®^ (Epic Systems Corp., Verona, WI, USA) interface with respect to the importance of anticoagulation treatment.

In contrast, the patients in the control group received only the standard care. These individuals also had access to the MyChart^®^ (Epic Systems Corp., Verona, WI, USA) interface, but did not receive training in its usage or the reception of customized interventions.

### Survey data and measures

Two surveys distributed at baseline and at the end of the study were created to (1) retrieve patients’ demographic information and perceived health status; (2) assess the level of medication knowledge about and the beliefs and attitudes regarding dabigatran usage; and (3) identify the degree of patient engagement. The survey was made available to patients in both print and online formats, using HIPAA-compliant software (SurveyMonkey^®^ San Mateo, CA, USA).

Participants’ medication knowledge was assessed via the presentation of five open-ended questions about dabigatran that focused on its indications, frequency of usage, mechanism of action, and potential adverse effects. The five questions can be found in **Table 1**, and were chosen as being crucial in terms of evaluating medication safety and the achievement of desired clinical outcome (ie, stroke prevention). The patients’ answers to the five questions were assessed by two pharmacy practice researchers. Correct answers were awarded one point, with a maximum obtainable score of five points. Kappa statistics were used to test inter-rater reliability. A high kappa coefficient (0.918, p < 0.05) ensures consistency between the researchers’ assessments. The differences of grading were re-evaluated by a clinical pharmacist to generate a final score for each patient.

Trends in the level of patient engagement of the study cohort were determined by the second survey administered, a validated 13-item survey instrument called the Patient Activation Measure^®^ (PAM^®^; Insignia Health, Portland, OR, USA).^20^ A list of the items in the PAM^®^ (Insignia Health, Portland, OR, USA) instrument can be found in **Table 2**. The PAM^®^ (Insignia Health, Portland, OR, USA) assesses an individual’s knowledge of and skill and confidence in managing their health. High scoring patients typically understand the importance of taking a proactive role in managing their health, and are generally equipped to do so. The PAM^®^ (Insignia Health, Portland, OR, USA) measures patients on a scale of 0 to 100, categorizing patients according to four activation levels along an empirically derived continuum. Patient engagement is measured by the extent to which a patient is involved in taking care of their health, and is critical in improving outcomes among patients with chronic health conditions. Our hypothesis sought to explore the impact of PHR use on patient activation, predicting higher PAM^®^ (Insignia Health, Portland, OR, USA) scores to correlate with improved medication adherence. PHR data were collected for each patient during the study. To determine the impact of varying PHR usage, all participants were categorized according to the number of times they logged into the PHR system over the course of the study’s three-month time period. Patients were designated as being of three user types: low (0–3 logins), active (4–9 logins), and super (10 + logins). These groups were determined based on breaks in the login frequency data, which helped to maintain an equivalent population size among the three types.

### Pharmacy refill data

Pharmacy refill data for dabigatran prescriptions were used to determine medication adherence. We utilized this longitudinal pharmacy data to calculate medication possession ratio (MPR) as a measure of adherence. The MPR was calculated as the sum of the days’ supply for all dabigatran refills during the study period divided by the number of days that elapsed during the study period. The range of days’ supply of dabigatran prescription was from 30 days to 90 days. Given the short duration of the study, only the 43 patients (48%) who refilled 30-day supplies for each prescription were included in the adherence calculation.

As reimbursement for patients’ time, effort and travel expenses, each individual received $20 after the completion of the first survey, and $50 after the completion of the second survey and submission of their prescription history data.

### Statistical analysis

Descriptive statistics were used to calculate the means and standard deviations for evaluating participants’ socio-demographic data. Paired t-tests were used to determine changes in medication knowledge and PAM^®^ (Insignia Health, Portland, OR, USA) scores before and after the study period. An independent group t-test was used to compare differences in medication adherence rates between the intervention and control groups. Spearman’s correlation coefficients were calculated to evaluate the associations between variables.^21^ The number of PHR logins was added to those variables to predict medication knowledge, adherence and PAM^®^ (Insignia Health, Portland, OR, USA) scores. SPSS version 22.0 (IBM Corp., Armonk, NY, USA) was utilized to analyze collected data.

## Results

**Table 3** reports on the sociodemographics of the study participants. The groups were similar in terms of baseline characteristics. The average age was 66 years, and the majority of the participants were Caucasian males. Most of the participants had a college or postgraduate degree. More than one-third of them had diabetes, and more than three-fourths had hypertension. At baseline, over half of the study participants rated themselves as good or very good with respect to their abilities to use a computer and the Internet, respectively. Almost all of the patients perceived their health status as either fair, good or very good, with only a few indicating they perceived themselves as having a poor health status.

### PAM^®^ outcomes

Forty-five patients in the intervention group and 41 patients in the control group completed the pre- and post-PAM^®^ (Insignia Health, Portland, OR, USA) survey. Both groups demonstrated similar PAM^®^ (Insignia Health, Portland, OR, USA) scores at baseline (63.03 ± 13.77 vs. 63.08 ± 14.73). However, the intervention group exhibited a slight increase in PAM^®^ (Insignia Health, Portland, OR, USA) scores across the study period (65.78 ± 13.92, p = 0.078), while the PAM^®^ (Insignia Health, Portland, OR, USA) scores in the control group stayed almost the same (63.56 ± 11.25, p = 0.814). There was no statistically significant difference in the change of PAM^®^ (Insignia Health, Portland, OR, USA) scores for either group.

### Medication knowledge

**Figure 3:**
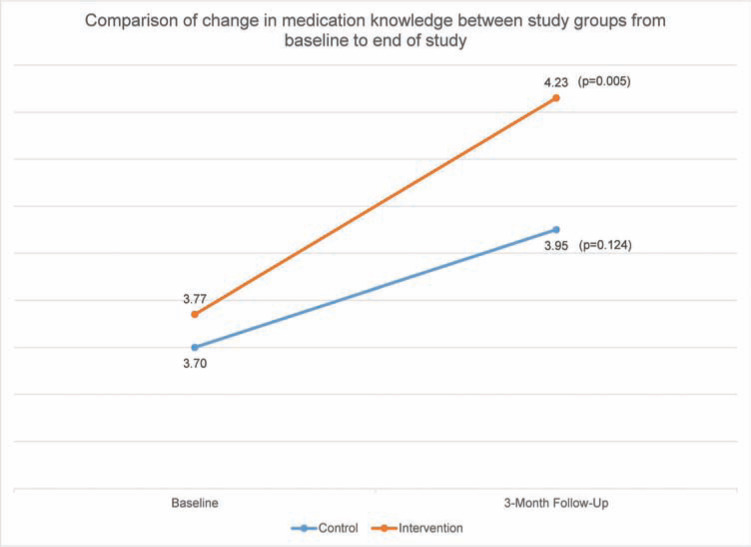
A comparison of the changes in medication knowledge between the study groups from baseline to end-of-study.

Forty-four patients in the intervention group and 40 patients in the control group completed the medication knowledge survey at both the time of enrollment and at the end of the study. Patients in the intervention group showed a significant improvement in their knowledge of dabigatran after the intervention (from 3.75 ± 0.892 to 4.32 ± 0.912, p = 0.005), as shown in **Figure 3**. The control group showed a slight increase in medication knowledge, though the increase was not statistically significant (from 3.70 ± 0.966 to 3.95 ± 0.846, p = 0.124).

### Medication adherence

**Figure 4:**
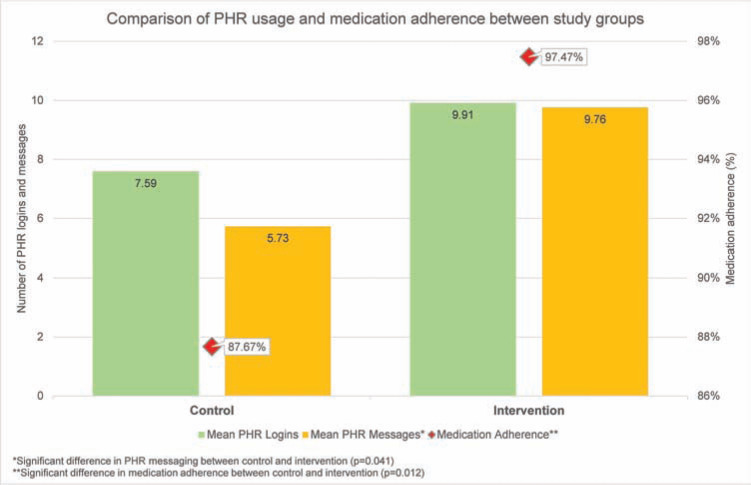
A comparison of PHR usage and medication adherence between the study groups.

All of the study participants successfully requested their dabigatran refill records from their pharmacy during the study period and returned them to research associates. Among these 90 refill records, 26 in the intervention group and 32 in the control group contained the necessary information about fill dates, quantity dispensed, days’ supply, and the medication strength that was required to calculate adherence. Since only patients filling 30-day supplies for each prescription were included in the adherence calculation, however, only 16 patients in the intervention group and 27 patients in the control group were included in the final adherence calculation, respectively. Although both groups demonstrated good dabigatran adherence rates by successfully exceeding the 80% adherence standard, the intervention group had significantly higher adherence rates as compared with the control group (97.47% ± 3.72 vs. 87.67% ± 14.48, p = 0.012), as shown in **Figure 4**.

### Correlation among PHR usage, medication knowledge and adherence

The mean number of PHR logins throughout the study was 8.78 ± 7.86. The intervention group demonstrated an average of 9.91 PHR logins, while the control group exhibited an average of 7.59 logins (p = 0.163). The study group utilized PHR messaging an average of 9.76 times, in comparison with the 5.73 times that the control group utilized PHR messaging (p = 0.041). There was a significant correlation between PHR usage and medication adherence (correlation 0.36, p = 0.018). Additionally, there was a significant relationship between patients’ pre-intervention medication knowledge and their level of medication adherence (0.369, p = 0.015). **Figure 4** depicts the trend in PHR usage between the study groups in relation with medication adherence.

## Discussion

To our knowledge, the current randomized controlled study that focused on anticoagulation therapy for AF patients is the first clinical investigation suggesting the positive impact of PHR use on medication management in clinical practice. In comparison with AF patients receiving standard care, patients who received the PHR interventions demonstrated a significant improvement in their knowledge of dabigatran, as well as higher medication regimen adherence. This research indicates that a PHR platform could be leveraged to improve health outcomes through utilizing available PHR features to conquer common barriers to medication adherence.

The PHR provides the potential to revolutionize the way patients and health care providers view responsibility regarding personal health. These systems facilitate a partnership between patients and physicians that could result in better health outcomes. PHRs can help to accomplish this by increasing patients’ understanding of their diseases and their participation in their own care, as they provide them with a tool they can use to communicate more effectively with their health care providers.

Patients’ beliefs and knowledge about the care of their health are key factors influencing their adherence to anticoagulant therapy and other medication regimens.^22,23^ Prior studies have revealed that many AF patients possessed limited knowledge of their disease, and did not sufficiently understand the risks and benefits of anticoagulant therapy.^24,25^ Lane et al. attempted to develop an intervention to improve patients’ knowledge of AF and anticoagulant therapy, demonstrating that a brief educational intervention with an information booklet can improve patient knowledge of anticoagulant therapy for AF.^26^ Our research indicates that adherence can be improved if patients know what they have been prescribed, the reason for taking it, possible side effects that may occur, and the implications of non-adherence.^27^ Our findings support the prior research by demonstrating that there is a significant relationship between medication knowledge and adherence.

Almost all interventions that lead to an effective improvement in chronic conditions are complex, including those combining more convenient care, information, reminders, reinforcement, counseling, family therapy, psychological therapy, telephone follow-up, and supportive care.^28^ While integrating all of the possible interventions into one platform has remained difficult, the PHR offers some potential as an inclusive tool that can be used to implement strategies to improve patients’ health behaviors.

Prior research had demonstrated a positive relationship between patient activation and PHR use. A 12-week study of patients with chronic diseases revealed improvements in patient activation and health outcomes associated with PHR use.^29^ Hibbard and Greene conducted a study of 16,357 patients who had recent primary care visits, and found that the more activated patients showed an increased likelihood of using a PHR.^30^ However, our research did not find a significant change in PAM^®^ (Insignia Health, Portland, OR, USA) scores in either group, nor did it observe a relationship between these scores and PHR use. Our results are comparable to those from a study performed by Ancker et al., who found that patients using a PHR (n = 180) were not more highly activated than nonusers, but were more educated and more likely to use the Internet.^31^ Similarly, Wagner et al. studied the use of PHRs to promote self-health management in hypertensive patients and found that the use of PHRs did not increase patient activation.^32^

### Study limitations

The applicability of this study to other regions of the US and other populations remains to be assessed. This study’s site was a single health care provider in Indiana. Our study’s participants were predominantly Caucasian males over 65 years of age who were educated, insured and living above the poverty level. While the age structure of this study population is representative of the AF population, the education levels and racial diversity seen in this study are not accurately representative of the US population.^33^ Further, the population for this study only included patients who had activated a PHR account for entry into the study. This requirement may have excluded individuals of lower socioeconomic levels who did not have access to the necessary technology, and may also have prevented older patients who do not utilize a PHR from participating. Therefore, our study’s population may be biased in this respect. Though 21.1% of our patient population was 75 years of age or older, the impact of the interventions on medication adherence in this older population group remains to be assessed. Our previous work, however, has demonstrated that the adoption of PHR technology by older patients can be enhanced with onsite training, and that this population can become super users.^19^

Ambiguity in answering questions pertaining to knowledge of anticoagulation with dabigatran was adjudicated by three experienced pharmacists from written and recorded interviews. However, this method may have some limitations, including restricting the number of questions to five and allowing for greater flexibility in responses (due to the open-ended nature of the questions).

Another limitation is that the use of medication refill history in this study’s regard is not as precise as the use of monitoring drug-dispensed medications with pill counts, since it does not permit observation as to whether patients have taken all of the dispensed dabigatran. However, using pharmacy dispensing data has been recognized as a reliable method to estimate adherence.^34^ Applying the same assessment method to both study groups also supports the validity of the comparison of medication adherence.

A final limitation that should be noted is that this study was conducted within a relatively short, four-month time frame. The initial enrollment period took place in February 2014, during a particularly harsh winter for the area, which could have made traveling to the study hospital for consent and training more difficult. Additionally, many residents prefer to spend their winters in warmer climates, which may have had an impact on the size and diversity of early recruitment. These factors can be linked to the relatively small sample size for the study. It is recommended that future studies take such factors into consideration.

## Conclusions

This study has implications for health policy leaders and health care providers. This is the first adherence study to confer an improvement on medication knowledge and adherence supported by PHR use. It also demonstrates the value of leveraging the PHR infrastructure that is now pervasive in most health systems and clinics, and provides a platform for improving patient care. If the anticoagulation therapy needs to be taken on a long-term basis, then optimal adherence is crucial in the prevention of stroke and in improving health-related outcomes.

## Acknowledgments

The authors would like to thank Tammy Toscos, MD, PhD, and Sarah Ellsworth-Hoffman, MLS, for their outstanding editorial review; and Riddhi Doshi, MPH, MBBS, for her significant contribution to the study’s design and development.

## Figures and Tables

**Table 1:** Survey Questions On Dabigatran Use Administered to Study Participants

(1)	Do you know why you were prescribed Pradaxa^®^ (dabigatran)?
(2)	How do you think Pradaxa" (dabigatran) works?
(3)	How often do you need to take Pradaxa" (dabigatran)?
(4)	Your nurse or physician may have explained to you that if a particular symptom appears while you are taking Pradaxa" (dabigatran), you should call your doctor right away. Do you know which symptom that is?
(5)	Do you need to get your blood tested regularly to monitor Pradaxa" (dabigatran)?

**Table 2:** Description of the 13-item Patient Activation Measure^®^ (PAM^®^) Survey20*

Patient Activation Measure^®^ (PAM^®^) Items**	
1.	When all is said and done, I am the person who is responsible for taking care of my health.
2.	Taking an active role in my own health care is the most important thing that affects my health.
3.	I am confident I can help prevent or reduce problems associated with my health.
4.	I know what each of my prescribed medications do.
5.	I am confident that I can tell whether I need to go to the doctor or whether I can take care of a health problem myself.
6.	I am confident that I can tell a doctor concerns I have even when he or she does not ask.
7.	I am confident that I can follow through on medical treatments I may need to do at home.
8.	I understand my health problems and what causes them.
9.	I know what treatments are available for my health problems.
10.	I have been able to maintain (keep up with) lifestyle changes, like eating right or exercising.
11.	I know how to prevent problems with my health.
12.	I am confident I can figure out solutions when new problems arise with my health.
13.	I am confident I can maintain lifestyle changes, like eating right and exercising, even during times of stress.

*PAM^®^ is a product of Insignia Health, Portland, OR, USA.

**Response options per item are four-point Likert scales, ranging from strongly disagree to strongly agree and “not applicable.”

**Table 3:** Demographic Data Comparison of Study Subjects Based on Group Assignment (n = 90)

Characteristic	Intervention Group (n = 46)	Control Group (n = 44)	p-Value
Characteristic	Intervention Group (n = 46)	Control Group (n = 44)	p-Value
Age (years), mean ± SD	66.1 ± 8.36	67.3 ± 9.1	0.503
Female gender	14 (30.4)	13 (29.5)	0.940
Ethnicity (Caucasian)	45 (97.8)	43 (97.7)	0.494
Ethnicity (other)	1 (2.2)	1 (2.3)	
Education			
High School and below	15 (36.9)	11 (27.3)	0.727
College degree	24 (52.2)	22 (50.0)	
Postgraduate degree	5 (10.9)	10 (22.7)	
Diabetes	20 (43.5)	15 (34.1)	0.361
Hypertension	39 (84.8)	38 (86.4)	0.832
Perceived health (good/very good)	24 (52.2)	28 (63.7)	0.361
Computer efficacy (good/very good)	24 (52.1)	24 (55.9)	0.754
Internet efficacy (good/very good)	25 (54.3)	24 (57.1)	0.560

n: number; SD: standard deviation.
